# The Potential of NGTs to Overcome Constraints in Plant Breeding and Their Regulatory Implications

**DOI:** 10.3390/ijms262311391

**Published:** 2025-11-25

**Authors:** Franziska Koller

**Affiliations:** Fachstelle Gentechnik und Umwelt (FGU), 80807 Munich, Germany; info@fachstelle-gentechnik-umwelt.de

**Keywords:** new genomic techniques (NGTs), genetically engineered organisms, genome editing, CRISPR/Cas, recombinant enzymatic mutagens (REMs), GMO regulation, risk assessment, process-oriented approval process

## Abstract

Conventional plant breeding relies on the occurrence of chromosomal crossover and spontaneous or non-targeted mutations in the genome induced by physical or chemical stressors. However, constraints exist concerning the number and variation of genotypes that can be achieved in this way, as the occurrence and combination of mutations are not equally distributed across the genome. The underlying mechanisms and causes of reproductive constraints can be considered the result of evolution to maintain the genomic stability of a species while at the same time allowing necessary adaptations. A continuous horizon scan was carried out to identify plants derived from new genomic techniques (NGTs), which show that CRISPR/Cas is able to circumvent at least some of these mechanisms and constraints. The reason for this is the specific mode of action: While physico-chemical mutagens such as radiation or chemicals merely cause a break in DNA, recombinant enzymatic mutagens (REMs), such as CRISPR/Cas, additionally interfere with cellular repair mechanisms. More recently developed REMs even expand the capabilities of NGTs to introduce new genetic variations within the target sequences. Thus, NGTs introduce genetic changes and combinations that are unknown in the current breeding pool and that are also unlikely to occur as a result of any previously used breeding methods. The resulting genotypes may need to be considered as ‘new to the environment’. The technical potential of NGTs should also be taken into account in regulatory provisions. Previously unknown genotypes and phenotypes may negatively impact plant health, ecosystems, biodiversity, and plant breeding. It must further be acknowledged that the different outcomes of NGTs and conventional breeding are not always evident at first sight. As a starting point, within a process-oriented approval process, molecular characterization can inform the following steps in risk assessment and guide requests for further data.

## 1. Introduction

Genome flexibility allows plants to evolve over generations and adapt to changing environmental conditions [1]. At the same time, genome stability is crucial to ensuring integrity and maintenance [2,3]. Plants have developed an extensive array of mechanisms and structures to cope with DNA damage and ensure proper cell division and genetic exchange [4,5]. These mechanisms and structures shape the occurrence and combination of mutations, which are not equally distributed across the genome [6].

Conventional plant breeding also works within this framework, as it relies on the occurrence of crossovers, as well as spontaneous or non-targeted mutations induced by physical or chemical stressors. Therefore, constraints exist for the number and variation of genotypes that can be achieved in this way.

Genome editing is a site-directed process that enables the editing of DNA sequences using recombinant enzymatic mutagens (REMs), such as clustered regulatory interspaced palindromic repeats/CRISPR-associated nucleases (CRISPR/Cas), transcription activator-like effector nucleases (TALEN), or zinc finger nucleases (ZFN). These techniques are referred to as new genomic techniques (NGTs). The application of NGTs allows genotypes and phenotypes/traits to be modified within short periods of time. While some of the NGT-derived genotypes are also achievable using conventional breeding, other genotypes are novel and go beyond what can be expected from conventional breeding [7,8,9,10,11]. For example, specific regulatory units in plant DNA are a highly dynamic research field; it is here that even minor indels (insertions or deletions) are often sufficient to produce significant impacts on gene expression [12,13,14]. However, many of these specific minor genetic changes achieved by NGTs seem to be hardly achievable with conventional plant breeding techniques. In the future, one can expect to see even more novel and increasingly complex genotypes driven by ongoing technical development, increasing knowledge of plant genetics, and diverse applications (see, e.g., [15]).

In the following sections, this review reports findings that explain the differences between NGTs and conventional breeding based on the mode of action of CRISPR/Cas nucleases (Section 2.1), cytogenic factors that influence the likelihood of mutations occurring in specific genic regions (Section 2.2.1), factors influencing recombination and stability of the genome (Section 2.2.2), and the role of gene copies (Section 2.2.3). In addition, this review provides examples that can be useful in discussions on the potential of NGTs and the differences from conventional breeding (Section 4).

Finally, implications of the findings in regard to the regulation and risk assessment of NGT plants are discussed.

## 2. Main Section: CRISPR/Cas-Catalyzed Reactions Can Overcome Structural Genomic Elements, Cytogenic Factors, and Mechanisms

### 2.1. Mode of Action of CRISPR/Cas

CRISPR/Cas was first discovered as an adaptive immune system in bacteria [16,17] and successively adapted as a molecular biotechnology tool for genome editing.

CRISPR/Cas is first and foremost a large enzyme complex that can be adapted for different purposes. Currently, CRISPR/Cas9 is the most used tool for altering plant genomes with site-directed nucleases (SDNs) since it is, amongst other things, easy to use and highly versatile. It consists of a targeting component, i.e., the single guide RNA (sgRNA) and the endonuclease Cas9. The endonuclease is a REM that can induce double-strand breaks (DSBs) in the DNA, which is the substrate, and thus trigger and interfere with various repair mechanisms in the cells, i.e., non-homologous end joining (NHEJ), microhomology-mediated end joining (MMEJ), and homology-directed repair (HDR), which repair the induced DSBs.

The NHEJ pathway is active throughout the cell cycle and ligates the two broken DNA ends without any homologous repair template. The outcome of NHEJ pathways may be either a DNA sequence that is identical to the one before the DSB, or a sequence with small insertions/deletions (indels) [18]. These indels can have significant effects, such as frameshift mutations, disruptions/alterations of gene functions, or changes in gene expression. Similarly to NHEJ, the MMEJ pathway does not require a template to repair the DSB. Instead, the DSB ends are trimmed until short homologous sequences, called microhomologous sequences, are exposed. This can result in very large deletions, as the MMEJ mechanism removes all sequences between the breakpoint and the microhomologous sequence. MMEJ is therefore regarded as highly mutagenic [19,20,21,22]. MMEJ activity is significantly elevated in actively cycling cells, i.e., when they enter the S and G2 phase [23]. It appears that MMEJ repair plays an important role in the repair during genome editing, as DSBs induced via Cas9 through enzymatic activity are more often repaired by MMEJ than DSBs caused by more unspecific stressors, e.g., radiation [3,24,25,26]. The HDR pathway uses homologous sequences from sister chromatids as a repair template, and it is therefore more accurate in repair than NHEJ or MMEJ. It is also primarily active in the S and G2 phase of the cell cycle when sister chromatids are available to be used as homologous templates [27,28].

In most cases of genome editing, these cellular repair mechanisms are used to disrupt and knock out gene functions by inducing DSBs (SDN1). However, in SDN2 and SDN3 approaches, the HDR pathway can be utilized to substitute, insert, or replace DNA sequences at the target site in a desired way using exogenous DNA donor templates [29,30,31].

CRISPR/Cas-catalyzed reactions specifically allow certain genomic regions (target sequences) to be cut with the endonuclease Cas9 using one or more sgRNA(s). The sgRNAs are designed individually according to the genomic loci that are to be altered. Cas9 interacts with the sgRNA and is directed to the DNA sequence complementary to the sgRNA [32,33]. The actual binding of the Cas9 to the DNA and its activation further require the presence of a protospacer adjacent motif (PAM) sequence adjacent to the target sequence [34]. After establishing the DNA/sgRNA/Cas9 complex, Cas9 then introduces a DSB at the target sequence (for more details see, e.g., [35]).

CRISPR/Cas9 mainly induces blunt-end DSBs [32,35,36], but according to a recent study, staggered ends can also occur in plants [37]. Depending on the time Cas9 stays bound to the cleaved products, it can smooth staggered ends after DSB [38,39]. Post-cleavage residency of the Cas9 enzyme can last several hours [39,40,41,42]. It is assumed that the release of Cas9 from the cleavage products is facilitated via certain cellular mechanisms rather than spontaneous dissociation [39,43]: The physical collision with enzymes of DNA replication and transcription can, for example, dislodge Cas9 from cleaved DNA ends [43,44,45]. Depending on the dissociation form, these cleaved ends may have different configurations, which in turn influence the result of the repair [43].

**Figure 1:**
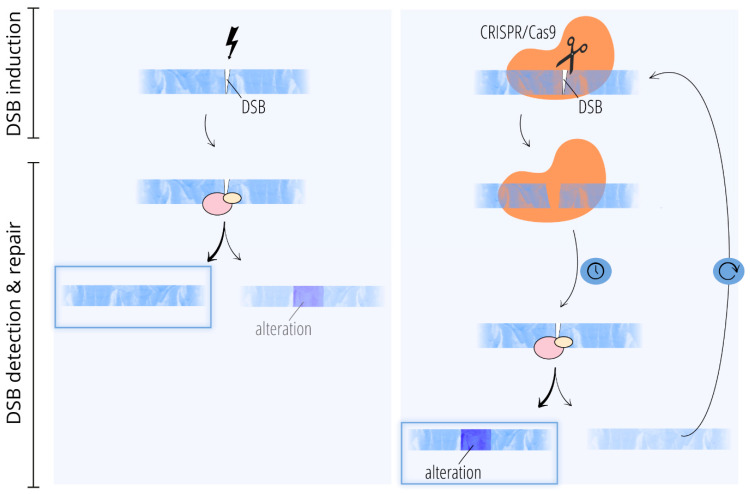
Repair of double-strand breaks (DSBs) without (**Left**) and with (**Right**) recombinant enzymatic mutagens (REMs). (**Left**) The DSB that occurs spontaneously or is induced in a non-targeted way by physical or chemical stressors is detected and repaired by repair proteins (red and yellow circles). Either the previous state is restored or the sequence is altered. (**Right**) After the induction of a DSB, Cas9 stays bound to the cleaved ends until the enzyme is, e.g., dislodged from the DNA. DSB detection, processing, and repair are therefore delayed compared to otherwise introduced DSBs. If the previous state of the DNA sequence is restored by the DNA repair process, the enzyme CRISPR/Cas will again catalyze the reaction and ultimately force a change. (**Left**, **Right**): In both cases, the more likely outcomes are indicated by blue frames.

However, as long as Cas9 is bound to the cleaved DNA, repair of the DSB cannot take place [43,44]. Therefore, cellular detection, processing, and repair of Cas9-induced DSBs is delayed compared to DSBs induced by e.g., radiation [40,41,43,46] (Figure 1). Further, it is assumed that the residency of the Cas9 enzyme and the delay have an impact on the outcome of the subsequent repair [43,47]. This may be one of the reasons why the repair of CRISPR/Cas9-induced DSBs is more error-prone and not representative of spontaneous, naturally occurring, or otherwise induced DSBs [40,41,46,47,48]. The extent of the error-prone repair is stated differently in the literature, but the results are confirmed in key findings [41,49]. The rates of error-prone repair seem to be locus-dependent, and further influencing factors remain to be investigated [41,49]. Once the ends of the DSB are released from Cas9, they can be detected and accessed by DNA repair machinery.

Overall, our understanding of CRISPR/Cas mechanisms and the subsequent repair of DSBs in the cell is incomplete [37]. However, studies have demonstrated that enzymatically induced DSBs with Cas9 are distinct from DSBs induced by, for example, ionizing radiation [43].

If the substrate (i.e., the binding site) is restored by DNA repair mechanisms, the enzyme CRISPR/Cas will again catalyze the reaction and ultimately force a change, as long as it remains active in the cell (Figure 1). In contrast, a mutation caused by physicochemical mutagens can also be repaired in such a way that the previous state is restored. As a result, CRISPR/Cas9 mutagens ultimately enrich mutagenic outcomes [41,50]. As Feng et al. state, the cell’s own DNA damage response and repair machineries have to respond to unique features of DSBs induced by Cas9 [43]. And in the context of medical research, Vitor et al. state the following: “*Moreover, the fact that Cas9-induced DSBs may be particularly refractory to repair, and hence biased in terms of repair pathway choice, call for caution when using these systems*” [51].

Several findings regarding CRISPR/Cas9-induced DSBs are derived from mammalian cells, but some findings are similarly relevant for NGT applications in plant cells [39]. For example, some studies reported an increase in chromothripsis due to CRISPR/Cas9 applications in mammalian cells [52,53,54,55]. Chromothrypsis is a catastrophic event resulting from the formation of DSBs on one or a few chromosomes and subsequent error-prone repair, generating highly rearranged chromosomes. The finding was explained to be related to the disturbed or delayed repair of DSBs ([56]; see also recently [57]), which is in line with the specific mode of action as reported above. Meanwhile, NGT-induced chromothrypsis was also reported in plants [58]. There are other examples of unintended effects of CRISPR/Cas9 that may be caused by its specific mode of action [59]. One specific effect is bi-allelic deletion inversions (delinvers), which can occur when CRISPR/Cas is used to create knockouts of tandemly arrayed genes [60,61] and would not be expected to occur as an outcome of conventional breeding.

### 2.2. Potential of NGTs to Overcome Structural Genomic Elements, Cytogenic Factors, and Mechanisms

#### 2.2.1. Cytogenic Features

In the past, evolutionary biology assumed that (i) the mutation rate is unbiased, i.e., it varies randomly among genomic loci and irrespective of the fitness consequence, and (ii) that the occurrence of mutations is strongly influenced by selection [62].

Both assumptions were called into question by the findings of Monroe et al., showing that mutation rates in *Arabidopsis thaliana* are influenced by cytogenic features and that the probability of de novo mutations is dependent on gene function and fitness consequences [63]. Prior studies already indicated a ‘mutational bias’ that is mediated by the DNA sequence itself, epigenetic features, and (the targets of the) various DNA repair mechanisms [64,65,66,67,68]. Accordingly, the former view of mutations in organisms as occurring entirely randomly cannot be upheld. The processes and mechanisms behind this observation can be considered the outcome of an evolutionary process to maintain genomic stability of the species while at the same time enabling necessary adaptations [6].

In detail, a strong bias in the mutation rate was demonstrated: The highest mutation rate was found in genomic regions carrying cytosine methylation and the histone modification H3K9me1 [63]. It is lower in guanine–cytosine (GC)-rich regions [63,65,69,70] and reduced inside genes [63]. Regions containing histone modifications (H3K4me1, H3K27Ac, and H3K36me3) that are enriched at actively transcribed genes show fewer mutations [63,71]. Other studies also found lower mutation rates in actively transcribed genes [66,72,73]. Since actively transcribed regions are more exposed to DNA damage, because they are less condensed and are sites of DNA processing [74], the observed low mutation frequency indicates that DNA repair mechanisms are especially active and efficient in highly transcribed regions [68,74]. Another major finding of Monroe et al. is that lower mutation rates correlate with essential genes that are enriched in epigenomic features associated with low mutation (e.g., H3K4me1, H3K36me3) [63].

These findings show that mutation rates across the genome depend on different cytogenetic features, including GC content, DNA methylation, histone modifications, chromatin accessibility, gene expression, and DNA repair mechanisms [7]. The latter is thought to be directed to (essential) genes, e.g., via H3K4me1 or H3K36me3 binding, which regulate the DNA repair machinery, i.e., HR, NHEJ, or DNA mismatch repair (MMR) [75,76]. Belfield et al. showed that MMR preferentially protects the genic parts of the genome (untranslated regions, coding sequence, and introns) from de novo mutations in *A. thaliana* [68,77]. There is also corresponding evidence in *Escherichia coli*, yeast, and human cell lines [78,79,80,81,82,83].

Thus, mutations are unevenly distributed across the *A. thaliana* genome [7]. Similar patterns were found in wheat and rice after chemical and radiation mutagenesis [6,74,84,85]. It is also well known that non-targeted mutagenesis with ethyl methanesulfonate (EMS) often results in multiple mutated alleles for one gene, but none for other genes [84].

##### Different Causes of Mutations Can Result in Different Outcomes

Comparing mutations caused by physico-chemical stressors with those caused by enzymatic site-directed nucleases is challenging: The editing efficiency and the outcome of CRISPR/Cas9 applications also seem to be influenced by cytogenic features [86,87,88]. For example, editing with CRISPR/Cas9 is less efficient in most types of heterochromatin compared to euchromatin, although certain heterochromatin features seem to promote highly mutagenic MMEJ [89,90]. In addition, several studies show that editing heterochromatin regions tends to be less effective but possible [39,88,91,92,93]. Further, other tools such as zinc-finger nucleases can edit genome regions that are otherwise inaccessible [87].

**Figure 2:**
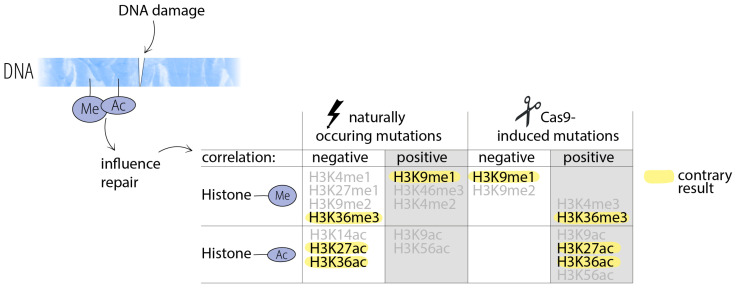
Histone modifications can influence the outcome of the DNA repair after damage. The repair of DNA damage that occurs spontaneously (table: left) or that is CRISPR/Cas9-induced (table: right) correlates with different histone methylations (Me) and acetylations (Ac). Contrary results are highlighted in yellow. Based on data published by Weiss et al. 2022 [88] and Monroe et al. 2022 [63].

As described above, studies found few mutations in actively transcribed regions when they occurred naturally or were otherwise induced. This was different from CRISPR/Cas-induced mutations: A relatively large number of mutations were found in actively transcribed genes of mammalian cells [44]. The authors could show that the RNA polymerase dislodges CRISPR/Cas9 from the cleaved DNA, where it was still bound. The collision with RNA polymerase converted Cas9 into a multi-turnover nuclease, resulting in repeated cutting and increased mutagenesis [44]. A detailed study in *A. thaliana* revealed further details of how various chromatin features influence Cas9 efficacy and the outcome of the DNA repair [88]. They observed, e.g., strong positive correlations between mutagenesis frequency and histone modifications H3K36ac, H3K27ac, and H3K36me3 after CRISPR/Cas9 interventions. This is in stark contrast to observations in *A. thaliana*, where the same histone modifications were correlated with fewer mutations when they occurred naturally [63] (Figure 2). These results strongly indicate that CRISPR/Cas9 is able to circumvent mechanisms in the cells that may otherwise protect certain areas of the genome. It can be concluded that CRISPR/Cas9 is a powerful tool that enables enzymatic alterations in the genome that would be effectively unachievable with conventional breeding.

#### 2.2.2. Factors Influencing Recombination and Stability of the Genome

**Figure 3:**
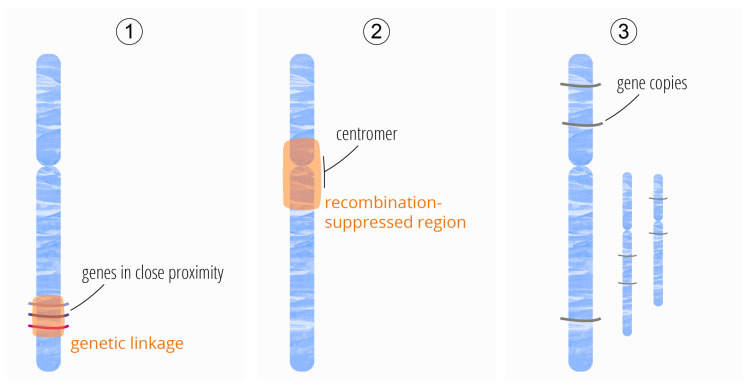
Constraints in conventional plant breeding: (**1**) genetic linkage, (**2**) recombination-suppressed regions, and (**3**) gene copies.

The genome of plants is organized into chromosomes of varying sizes and numbers. During meiosis, the chromosomes provide structure for genetic linkage groups, as genes or sequences that lie on the same chromosome are inherited as a group [94]. Recombination can separate genes or other DNA sequences on the same chromosome during meiosis. This requires an initial meiotic DSB and a subsequent crossover repair, resulting in an exchange of genetic material across homologous chromosomes. Hundreds of DSBs are induced during meiosis, but only very few result in crossovers [95,96]. The meiotic DSBs and crossover events seem to occur preferentially at narrow hotspots probably defined by a combination of DNA sequences and epigenetic factors [97,98]. In general, a crossover pattern is the outcome of a highly complex, regulated process, including genome-wide, chromosomal, and local regulation [96,99,100]. One result is that distant genes are more easily separated by crossover, whereas neighboring genes tend to be genetically linked. The latter group of genes is hardly accessible for breeders since they rely on natural meiotic recombination to generate new favorable allelic combinations, known as ‘linkage drag’ (Figure 3(1)).

Some genomic regions are strongly crossover-suppressed (“cold regions”), including sex chromosomes and centromeres [98,101] (Figure 3(2)). The centromeres and the surrounding so-called pericentromeric regions are characterized by large arrays of repetitive sequences, which are integral to centromere function and stability [94,102]. Specific histone variants and post-translational modifications are enriched at these repetitive sequences [103]. Recombination was shown to be strongly suppressed in centromeric regions in different plant species [96]. In barley [104], cotton [105], maize [106], tomato [107], and wheat [108], there are additional large pericentromeric regions with a low or absent number of crossovers, while at the same time, a considerable number of genes are located within this region [96,104,109]. This lack of recombination results in strong genetic linkage, as genes that lie in centromeric or pericentromeric regions will most likely be inherited together, making these regions effectively inaccessible to breeders [96,110]. Different studies show that crossover suppression in the proximity of centromeres is important for fertility [111,112,113].

The mechanisms behind the suppressed recombination in centromeric and pericentromeric regions are not fully understood. In *Arabidopsis*, the DSB density was shown to be reduced in centromeric regions [114]. However, this is not sufficient for entirely explaining the strong suppression [96]. The repair of meiotic DSBs in centromeric regions may favor inter-sister or non-crossover repair pathways instead of crossover repair [115]. There is probably an interplay between different mechanisms and factors that suppress meiotic DSBs or crossover repair, namely the following: epigenetics (e.g., histone modifications such as H3K27me3 or H3K9me2), genetic variations (polymorphism), and centromere-specific protein complexes (e.g., the kinetochore) [115].

The distances between genes to be recombined by crossing over are measured in centimorgans and vary not only between plant species but also in plant populations within the same species [109]. Taken together, there is a fine balance between the occurrence and suppression of crossover events in number and position [96].

##### Recombinant Enzymatic Mutagens Can Create Novel Patterns of Crossovers and Bypass Genetic Linkage

REMs can, for example, bypass the genetic linkage of specific gene regions. Alterations are induced to circumvent genetic linkage of traits such as, e.g., tomatoes (see Chapter 3, [116]). This technical potential is highly relevant in plant breeding as many genes are associated with linkage drag [96,104,109,110]. For example, tandemly arrayed genes (TAGs) with functional redundancy and chromosomal linkage constitute ~14–35% in sequenced plant genomes [61].

Dissections of linked genes can also be achieved by technically induced crossovers: The use of CRISPR/Cas makes it possible to change these natural meiotic recombination patterns [117], thereby manipulating and guiding inheritance. Novel crossing-over patterns can also be achieved by targeted crossover or chromosome reconstruction. Both CRISPR/Cas-induced approaches are further explained below.

In contrast to natural crossover events, targeted crossover is mainly induced in somatic cells [118,119]. In this way, competition with naturally occurring crossover events can be avoided, as somatic crossover events occur at a much lower rate than during meiosis. CRISPR/Cas9 is used here to induce DSBs, which can be subsequently repaired by (rare) somatic homologous recombination [58,118,120,121]. This can finally result in somatic crossover and be transmitted to the next generations, but so far, the approach has shown low efficiency in *A. thaliana*, tomato, and maize [15,58]. Unintended chromosomal loss and major chromosomal rearrangements (chromothripsis) were detected in addition to somatic crossover, which had various deleterious effects on the plant [58]. Nevertheless, there were some indications of successful induction of somatic homologous recombination in “cold regions” [121].

Furthermore, chromosome reconstruction can be used to manipulate genetic exchange when genetic linkages are broken or established [122]. The physical separation of two target genes, which naturally lie in close proximity, can break natural genetic linkage, whereas the combination of target genes on the same chromosome in close proximity can establish genetic linkages. In addition, repositioning a gene from a “cold region” might make it accessible for the exchange, e.g., of pericentromeric regions into an euchromatic chromosome arm environment [15,123]. This can be achieved by changing the order of genes on a chromosome (e.g., inversions) or by reciprocal translocation, i.e., the exchange of parts between non-homologous chromosomes [122,124,125]. Using CRISPR/Cas, the simultaneous induction of two DSBs on the same chromosome can lead to inversions, whereas the induction of two DSBs on different chromosomes can lead to reciprocal translocation [125]. The use of an egg-cell-specific Cas9 expression can enable heritable translocations [119,124,125]. Induction of large inversions on chromosomes can result in strongly suppressed [117] or, in the case of reversion of naturally occurring inversions, increased recombination [126,127]. The technique of chromosomal reconstruction is still in its infancy, but it enables a completely new level of targeted chromosome engineering [15,122,128]. In accordance, Schmidt et al. point out that “many […] examples show the potential of targeted modification of chromosome structures for achieving new combinations of alleles out of a pool of species that are not achievable by the use of classical breeding” [129].

Using these various approaches, researchers expect to bypass the limitations of meiotic recombination [118,120]. Previous attempts have shown that it is possible to fix favorable traits by generating new genetic linkages, suppress the recombination of whole chromosomes, separate beneficial traits from undesirable traits linked to them, or unlock “cold regions” for genetic exchange [15,126]. Chromosomal rearrangements also occur in nature and can play an essential role in the speciation and chromosome evolution of plants [130,131]. However, chromosomal rearrangements like reciprocal translocation or inversions seem to occur quite rarely and are counter-selected [132,133]. In addition, chromosomal rearrangements may occur non-randomly in hotspots [134,135,136,137,138]. Growing evidence suggests that chromosomal rearrangements are frequently associated with heterochromatic regions composed of repetitive DNA sequences [3,139]. It is speculated that if those rearrangements occur in plants, they have a minimally deleterious impact on the genome, giving chromosomes a safe place to break or fuse [137,139]. Interestingly, transposon insertion also seems to be tightly correlated to epigenomic features [6,140,141].

Using CRISPR/Cas, it is already possible today, or will likely be possible in the near future, to induce genetic exchange at almost every position in the genome, thereby circumventing natural limitations/patterns of meiotic crossover. Targeted chromosomal reconstruction further enables a completely new level of genome engineering. Thereby, the mechanism of inheritance can be overcome, and genetic combinations can be achieved that would not occur naturally. In the future, researchers expect to achieve even more complex or fine-tuned rearrangements, such as promoter swapping, the construction of artificial centromeres, or mini cargo chromosomes on which beneficial genes can be stacked [15,142].

#### 2.2.3. Gene Copies with and Without Proximity

Gene duplication is common in all investigated species, but the rates are significantly higher in plants compared to most other eukaryotes [143]. Gene copies can result from ancient duplication events, such as whole-genome duplication (polyploidization), chromosome and subchromosomal duplication (segmental duplication), or local duplication (tandem duplications) [144,145]. This results in many gene copies: 65% of annotated plant genes have, on average, at least one copy [145]. Depending on how long ago a gene duplication occurred and how many spontaneous mutations have accumulated since then, a duplicated gene can either retain its function so that the gene is redundant or develop into a gene with split or new function (sub- and neofunctionalization). Some duplicated genes are located in close proximity (e.g., gene clusters such as tandemly arrayed genes), whereas others are interspersed throughout the genome, depending on their origin and further genome evolution [145].

Using conventional plant breeding techniques, it is difficult, time-consuming, and in part impossible to achieve modifications or the knockout of all the different copies of a gene, especially if they are genetically linked, as in gene clusters [146] (Figure 3(3)).

Using CRISPR/Cas, it is possible to target and alter all copies or variants of a gene present in the genome, irrespective of how many gene copies are present [7,68]. CRISPR/Cas can also be used to target similar genes from gene families within conserved target sites [147]. In addition, it also enables the editing of different genomic sites using different sgRNAs in one organism simultaneously or successively in so-called multiplexing approaches [7,68]. These technical potentials may be especially relevant in plants with polyploid genomes such as wheat: for example, to achieve resistance to pathogens [148] or to reduce the concentration of certain proteins [149].

#### 2.2.4. Other Genomic Features

As shown in Section 3, there are examples of NGT plants with only minor indels in regulatory units resulting in new genotypes, e.g., lettuce [150] and strawberries [151]. These are previously unknown genotypes. Much of the current research is focused on regulatory units in plant genomes and their effects on the expression of endogenous genes. Attractive targets include small regulatory elements that impact gene expression, e.g., promoters [12], upstream open reading frames (uORFs) [13], and microRNA (miRNA) [14]. There are further examples of NGT plants with alterations in their regulatory elements, e.g., the deletion of an auto-inhibitory domain of a protein [152] or the knockout of a regulatory protein [153].

Such changes could theoretically also be expected to result from non-targeted mutagenesis. However, as can be assumed from the studies above, they were not known to previously exist within the breeding pool [150,151,152,153]. Regarding regulatory elements like promoters, the desired effects often require the introduction of several specific changes within the target sequence, while the deletion of the entire regulatory sequence would often destroy the three-dimensional genomic architecture [154]. Interestingly, current CRISPR/Cas9-induced mutations often failed to alter the promoter function significantly [154,155], but newly developed types of REMs were successful [155]. Thereby, the genome editing capabilities are further expanded and are going beyond ‘simple’ knock-outs [155]. New members of REMs are, e.g., CRISPR/Cas12a for larger deletions [154], various base editors for key nucleotide conversion [156,157], and prime editors for the substitution of small DNA sequences via reverse transcription using templates [155,156,158,159,160].

The specific technical potential of these REMs was also shown in the design of NGT plants developed by artificial intelligence (AI), going beyond what can be expected from conventional breeding [161].

## 3. Examples Showing the Potential of NGTs to Overcome the Constraints of Conventional Breeding

The use of NGTs can result in new genotypes that go beyond what can be expected from conventional breeding, including non-targeted mutagenesis [7,10,162,163]. To what extent REMs can overcome constraints in conventional breeding, i.e., whether the plant genome is totally available for any type of genetic manipulation, will naturally depend on the stage of advancement of NGTs.

This section presents examples demonstrating the current potential of NGTs, as well as the differences from conventional breeding. They are taken from the continuous horizon scan carried out by Project Genetic Engineering and the Environment (https://fachstelle-gentechnik-umwelt.de/en (accessed on 22 November 2025)) since 2019 and are based on the published literature and online databases for genome-edited crops. Table 1 contains a summary. The list below is not meant to be exhaustive, but it is intended to provide particularly clear examples of NGT-derived plants that would hardly have been achieved by conventional breeding.

### 3.1. Tomato with Improved Harvesting Properties and Plant Architecture

Breeders found a phenotype in wild tomatoes (*Solanum pimpinellifolium*) where the fruit does not detach from the plant (no pedicel abscission), a trait which is of interest for the tomato harvest [164]. Conventional breeding failed to cross this trait into currently marketed fresh tomato breeds, as it comes with unwanted traits such as undesirable fruit shapes [165]. Researchers later found a naturally occurring mutation in the gene jointless 2 (*j2*) responsible for the desired trait [116,166]. It is located close to the centromere of chromosome 12, an area with suppressed recombination, explaining the difficulty of separating the desired ‘jointless’ trait from the undesired traits due to genetic linkage [165,166]. Using CRISPR/Cas, researchers were able to incorporate the ‘jointless’ trait into marketed fresh tomato breeds [165]. In addition, by combining various CRISPR/Cas-induced mutations in *j2* and enhancer of jointless 2 (*ej2*) genes, the researchers were able to fine-tune the plant architecture and number of fruits to an unprecedented degree [116,167].

### 3.2. De Novo Domesticated Tomato

De novo domestication, i.e., the use of NGTs for a rapid conversion of wild relatives into crop plants [168], was performed in 2018 in the wild form of tomatoes (*S. pimpinellifolium*) [169]. The researchers simultaneously modified several genes in *S. pimpinellifolium*, thereby establishing agronomically desirable traits. This multiplex approach resulted in completely novel genotypes, which are not achievable with conventional breeding techniques, especially in crops such as tomatoes, where the linkage drag affects large parts of the genome [170]. There are further examples of multiplexing in tomatoes hardly achievable with conventional breeding, e.g., the (over-) accumulation of GABA, lycopene, and pigments (recoloring) [171,172,173].

### 3.3. Camelina with Altered Fatty Acid Content

In order to alter fatty acid contents in *Camelina sativa*, researchers targeted conserved genomic regions to knock out all three fatty acid desaturase genes (*fad2*) [174]. *C. sativa* is an allohexaploid plant, meaning that the genome is composed of three sub-genomes, and the genes of interest are present in several copies. It is extremely difficult to change several copies of a gene at different locations in the genome using conventional breeding, especially when the copies are located in different parts of the genome [175]. Other multiplexing approaches in camelina uncovered new flowering and architecture traits targeting up to 20 gene copies/alleles simultaneously [176].

### 3.4. Rice with Modified Flavone Content

Researchers knocked out two genes (flavonoid hydroxylases *cyp75b3* and *cyp75b4*) to produce a rice (*Oryza sativa*) with altered flavone content [177]. The genotype would be hardly achievable using conventional breeding methods, as the two targeted genes are genetically linked and probably lie in the pericentromeric region, where recombination is known to occur at a low rate [178,179,180,181,182,183,184,185,186].

### 3.5. Wheat with Low Gluten or Asparagine Content

In order to obtain wheat with low gluten content, researchers targeted conserved regions of α-gliadin genes in allohexaploid bread wheat (*Triticum aestivum*) with CRISPR/Cas9 [187]. They were able to simultaneously target up to 35 α-gliadins genes. In contrast, conventional breeding has failed to obtain comparable varieties due to high numbers of gene copies.

In addition, there are approaches to further reduce gluten content in wheat, where researchers target ω- and γ-gliadin gene clusters using CRISPR/Cas9 [188,189]. Crossing these lines with NGT lines, which already had 35 α-gliadin modifications, allowed the stacking of even more mutations [189]. According to Rottersmann et al., NGTs will be necessary to produce gluten-free wheat [149].

Other approaches strongly reduced the level of free asparagine in wheat. This was achieved by knocking out all six alleles of the asparagine synthetase gene (*asn2*) [190].

### 3.6. Rice with Low Glutelin Content

Using CRISPR/Cas9, researchers generated rice (*O. sativa*) with drastically decreased contents of major glutelins by targeting the five major glutelin genes *glua3*, *glub1a*, *glub1b*, *glub2*, and *gluc* [191]. In the past, breeders were already able to reduce glutelin content in rice; however, with CRISPR/Cas9, it became possible to achieve genotypes and phenotypes going beyond any prior breeding attempt. Several of the targeted glutelin genes are located in close proximity to each other and are organized in a gene cluster on chromosome 2. This genetic linkage is hardly accessible for conventional breeding.

### 3.7. Sugarcane with Less and Modified Lignin

In order to reduce both total lignin content and the ratio of syringyl to guaiacyl (S/G ratio), thus improving its suitability for use as a biofuel, researchers targeted the lignin biosynthetic caffeic acid O-methyltransferase (*comt*) genes in sugarcane (*Saccharum officinarum*) [192]. They used TALEN to target a conserved region in *comt* genes and achieved mutations in more than 100 *comt* copies/alleles. Modern sugarcane cultivars are highly allo-autopolyploid and have highly complex genomes with up to 130 chromosomes, resulting in a high level of genetic redundancy with around 12 homo(eo)logs at each locus [192,193]. Changes on this scale are hardly achievable with conventional breeding methods. This example shows that while CRISPR/Cas is very popular and frequently applied, other REMs, such as TALEN, are not outdated.

Meanwhile, further studies have shown the potential of CRISPR/Cas multiplexing to create novel genotypes and phenotypes in sugarcane, e.g., reduced chlorophyll contents, herbicide tolerance, and the above-mentioned low lignin content combined with high S/G ratios [194,195,196].

### 3.8. Switchgrass with Increased Tiller Production

Researchers targeted three genes (teosinte branched 1 (*tb1a* and *tb1b*) and phosphoglycerate mutase (*pgm*)) for increased tiller production in switchgrass (*Panicum virgatum*) [197]. Switchgrass is self-incompatible and a highly heterozygous polyploid (tetraploid and octoploid) species [198], resulting in severe restrictions for conventional breeding [199].

Researchers also demonstrated other approaches to increase tiller number by targeting further members of the tb1/cycloidea/proliferating cell factor gene family [200].

### 3.9. Tomato with Increased GABA Content

In a study from 2017, the autoinhibitory domain of two genes (Glutamate decarboxylase genes *gad2 and gad3*) in the γ-Aminobutyric acid (GABA) biosynthesis of tomato (*S. lycopersicum*) was successfully deleted [152]. A stop codon was introduced immediately upstream of the autoinhibitory domain in the C-terminus, which strongly increased the GABA content in tomato fruits. Interestingly, the authors also applied chemical mutagenesis that did not lead to comparable genotypes. Although they identified several mutations in the *gad2* and *gad3* genes, all of them were located around the N-terminus of the respective gene.

It is speculated that mutations in this particular part of the target genes may be naturally impeded by MMR activity [7].

### 3.10. Early-Flowering Poplar

CRISPR/Cas9 was used to radically shorten the juvenile phase in poplars (*Populus* spp.) from several years to just a few months [153]. They targeted a negative regulator of floral initiation, centroradialis (*cen1* and *cen2*), and a female-specific, type-A response regulator (*arr17*) to achieve novel phenotypes with early flowering, sex switch, and sex morphs. In nature, poplars have a juvenile phase of seven to ten years, resulting in long generation times, which is a major impediment to conventional breeding [201].

Previously, similar attempts in poplar trees using the insertion of transgenes were less effective and resulted in trees with first flowering just under three years [202].

### 3.11. Rice with Asexual Reproduction Enabling the Maintenance of Hybrids (Synthetic Apomixis)

Researchers were able to fundamentally change reproduction in rice (*O. sativa*) [203]. They targeted baby boom 1–3 (*bbm*1–3) genes leading to embryo arrest and abortion, which are fully rescued by the expression of male *bbm1* in egg cells. Further combination with a triple knockout of three meiotic genes (*rec8*, *pair1*, and *osd1*) induced mitosis instead of meiosis (MiMe) and resulted in synthetic asexual propagation. So far, this approach faces some problems, but it could potentially enable the maintenance of hybrids through synthetic apomixes and other approaches that have already been developed, including in maize (*Zea mays*) [204,205]. It can be reasonably assumed that such complex changes are impossible to achieve with conventional breeding.

### 3.12. Mustard Greens with Reduced Pungency

In order to reduce pungency in mustard greens (*Brassica juncea*), researchers targeted the type-I myrosinase multigene family using CRISPR/Cas12 [206]. They introduced mutations in all functional and expressed myrosinase genes simultaneously in 34 copies/alleles. *B. juncea* is an allotetraploid organism with a high number of duplicated genes, which leads to constraints in conventional breeding [207].

### 3.13. Maize with Increased Drought Tolerance

In order to increase the drought tolerance of maize (*Z. mays*), researchers developed a maize variant that overexpresses *argos8*, a negative regulator of ethylene response [208]. Using CRISPR/Cas9, they inserted a native maize promoter, which conferred a constitutive expression of the gene. The native promoter was either inserted in the 5′-untranslated region of the native *argos8* gene or swapped against the native promoter of *argos8*. This artificial transfer of sequences can be considered to be cisgenesis, as the promoter originates from the same species. Conventional breeding failed to achieve a phenotype with comparable expression of the *argos8* gene [208]. Although the transfer of desirable DNA sequences is theoretically possible with conventional breeding, the genotype is also unlikely to be achieved as transferred DNA normally drags large segments of flanking DNA [118].

### 3.14. Rice with Fine-Tuned Protein Expression

Researchers fine-tuned the expression of various genes in rice (*O. sativa*) by manipulating upstream open reading frames (uORFs). These regulatory elements repress the translation initiation of the downstream mRNA by sequestering ribosomes [209]. The researchers used base editing or prime editing to generate de novo uORFs, e.g., by inserting 1–3 bases to create upstream ATGs or to extend existing uORFs by mutating their stop codons. Other studies already introduced the knockout of uORFs using CRISPR/Cas, which increased protein expression [150,151,210].

Conventional breeding seems to be limited due to rare naturally occurring mutations in gene-regulatory regions [13,211]. In addition, it would be practically impossible to achieve the introduction of de novo uORFs using conventional breeding.

There are further specific NGT applications to interfere with regulatory units, such as miRNA (see, for example, [14]) or cis-regulatory elements and altered gene expression (see, for example, [12,155]) that result in genetic changes that could not be achieved with conventional breeding methods. These applications are based on more recently developed REMs that, for example, allow the ‘fine-tuning’ of gene expression in plants, going beyond ‘simple’ knockouts [155].

**Table 1:** Examples showing the potential of NGTs to overcome the constraints of conventional breeding.

Traits	Altered Gene(s)	Ploidy Level	Number of Genomic Alterations	Constraints for Conventional Breeding	Reference
**Bread Wheat** (*Triticum aestivum*)					
Reduction in gluten content	α-gliadins	hexaploid	up to 35 genes simultaneously	gene copies, genetic linkage (gene cluster)	Sánchez-León et al., 2018 [187]
Reduction in gluten content	ω- and γ-gliadins	hexaploid	up to 9 ω-gliadin and 12 γ-gliadin genes simultaneously	gene copies, genetic linkage (gene cluster)	Yu et al., 2023; Sánchez-León et al., 2024 [188,189]
Reduction in asparagine content	asparagine synthetase (*asn2*)	hexaploid	3 genes simultaneously in 6 alleles	gene copies	Raffan et al., 2021 [190]
**Camelina/false flax** (*Camelina sativa*)					
Early flowering, shorter stature, and/or basal branching	flowering locus c (*flc*), short vegetative phase (*svp*), like heterochromatin protein 1 (*lhp1*), terminal flower 1 (*tfl1*) and early flowering locus 3 (*elf3*)	hexaploid	up to 10 genes simultaneously in up to 20 alleles	gene copies, genetic linkage	Bellec et al., 2022 [176]
Reduction in polyunsaturated fatty acids and increase in oleic acid	fatty acid desaturase 2 (*fad2*)	hexaploid	up to 3 genes simultaneously in up to 6 alleles	gene copies	Morineau et al., 2017 [174]
**Lettuce** (*Lactuca sativa* L.)					
Increase in ascorbic acid content and oxidation stress tolerance	GDP-l-galactose phosphorylase (*ggp1* and *ggp2*)	diploid	1 gene in 2 alleles	rare naturally occurring mutations in gene-regulatory regions	Zhang et al., 2018 [150]
**Maize** (*Zea mays)*					
Increase in drought tolerance	*argoS8*	diploid	1 gene in 2 alleles	artificial transfer of cisgenic sequences	Shi et al., 2016 [208]
**Mustard Greens** (*Brassica juncea*)					
Reduction in pungency	type-I myrosinase multigene	allotetraploid	17 genes simultaneously in 34 alleles	gene copies, genetic linkage	Karlson et al., 2022 [206]
**Poplar** (*Populus* spp.)					
Early flowering, sex-switch, and hairless seeds	centroradialis (*cen1* and *cen2*), type-A response regulator (*arr17*), MYB transcription factors (myb186/138/38 (Fuzzy3))	diploid	up to 3 genes simultaneously in different combination of 1 arr17, 8 myb, and 4 cen1/cen2 alleles	Not known	Ortega et al., 2023 [153]
**Rice** (*Oryza sativa*)					
Reduction in glutelin content	glutelins (*glua3*, *glub1a*, *glub1b*, *glub2*, and *gluc*)	diploid	5 genes simultaneously in 10 alleles	genetic linkage (gene cluster)	Wakasa et al., 2024 [191]
Synthetic apomixes, maintenance of hybrids	baby boom (*bbm1*, *bbm2* and *bbm3*) and meiotic genes (*rec8*, *pair1* and *osd1*)	diploid	up to 6 genes simultaneously	complex multiplexing including expression of male-genome-derived BBM1 in egg cell	Khanday et al., 2019 [203]
Fine-tuning of gene expression	uORFs of various genes	diploid	1 gene	rare naturally occurring mutations in gene-regulatory regions, de novo sequences	Xue et al., 2023 [209]
Increase in apigenin content	flavonoid 3′ hydroxylases (*cyp75b3* and *cyp75b4*)	diploid	2 genes simultaneously in 4 alleles	genetic linkage, centromere/suppressed recombination	Yan et al., 2022 [177]
**Strawberry** (*Fragaria vesca*)					
Increase in sugar content	transcription factor basic (region) leucine zipper proteins (*FvebZIPs1.1*)	diploid	1 gene in 2 alleles	rare naturally occurring mutations in gene-regulatory regions	Xing et al., 2020 [151]
**Sugarcane** (*Saccarum officinarum*)					
Reduction in lignin content and syringyl/guaiacyl (S/G) ratio	caffeic acid O-methyltransferases (*comt*)	allopolyploid	107 alleles simultaneously	gene copies	Kannan et al., 2018 [192]
Reduction in chlorophyll content	magnesium chelatase subunit I (*mgch*)	allopolyploid	49 alleles simultaneously	gene copies	Eid et al., 2021 [194]
Herbicide tolerance	acetolactate synthase (*als*)	allopolyploid	3 alleles simultaneously	gene copies	Oz et al., 2021 [195]
Reduction in lignin content and increase in S/G ratio	transcription factor LIM (*lim*)	allopolyploid	not specified	gene copies	Laksana et al., 2024 [196]
**Switchgrass** (*Panicum virgatum*)					
Increase in tiller production	teosinte branched 1 (*tb1a* and *tbtb*) and phosphoglycerate mutase (*pgm*)	heterozygous polyploid	up to 2 genes simultaneously with multiple alleles for each gene	gene copies, self-incompatible	Liu et al., 2018 [197]
Increase in tiller production	tb1/cycloidea/proliferating cell factor (*tcp19* and *tcp 22*)	heterozygous polyploid	2 genes simultaneously in 4 alleles	gene copies, self-incompatible	Sun et al., 2025 [200]
**Tomato** (*Solanum lycopersicum*)					
Increase in GABA content	tomato phytoene desaturase (*slyPDS*), pyruvate-dependent GABA-T (*gaba-tp1, gaba-tp2* and *gaba-tp3*), transporter cat9 (*cat9*) and Succinate semialdehyde dehydrogenase (*ssadh*)	diploid	up to 4 genes simultaneously in up to 8 alleles	genetic linkage	Li et al., 2018 [171]
Increase in lycopene content	cyclisation of lycopene (*lcy-e, lcy-b1, lcy-b2,* and *blc*)	diploid	up to 4 genes simultaneously in up to 8 alleles	genetic linkage	Li et al., 2018 [172]
Accumulation of pigments	phytoene synthase 1 (*psy1*), R2R3-MYB transcription factor (*myb12*), stay-green 1 (*sgr1*)	diploid	3 genes simultaneously in 6 alleles	genetic linkage	Yang et al., 2023 [173]
Increase in γ-aminobutyric acid (GABA) content	glutamate decarboxylase (*gad2/3*)	diploid	2 genes simultaneously in 4 alleles	specific alteration in regulatory domain	Nonaka et al., 2017 [152]
jointless trait, floral architecture	jointless 2 (*j2*), weak enhancer of jointless 2 (*ej2*)	diploid	up to 2 genes simultaneously in up to 4 alleles	genetic linkage, centromere/suppressed recombination	Roldan et al. 2017, Soyk et al., 2017, Klee 2019 [116,165,166]
**Tomato** (*S. pimpinellifolium*)					
De novo domestication	self-pruning (*sp*), ovate (*o*), fruit weight 2.2 (*fw2.2*), lycopene beta cyclase (*cycb*), fasciated (*fas*)/clavata 3 (clv3), multiflora (*mult*)	diploid	up to 4 genes simultaneously in different homozygous and heterozygous allele combinations	genetic linkage	Zsögön et al., 2018 [169]

## 4. Discussion: The Relevance of Differences Between NGTs and Conventional Breeding for Risk Assessment and Regulation

The examples presented above show that many of the constraints impacting crossing and selection, as well as the outcomes of physicochemical mutagenesis, are not valid when recombinant enzymatic mutagens (REMs) are applied. Some NGT plants may nevertheless have similar genotypes and phenotypes to those obtained from conventional breeding [212]. Therefore, the regulatory framework for NGT plants should be guided by the comparison with breeding techniques that have a history of safe use and are not subject to mandatory risk assessment [213].

### 4.1. Comparison with Conventional Breeding

Conventional breeding is generally considered to introduce traits by crossing and selection, which results in new genetic combinations. It can also enrich genetic diversity through the use of physicochemical stressors in non-targeted mutagenesis [214].

In comparison to conventional breeding (physicochemical mutagens), NGTs do not introduce new types of mutations, i.e., genetic modifications, which can be categorized as insertions, substitutions, deletions, and inversion [215]. Furthermore, the number of mutations is typically no higher than those obtained from non-targeted mutagenesis [216,217,218].

However, as shown above, NGTs can overcome several of the known constraints in conventional breeding, i.e., cytogenic features, factors influencing recombination and stability of the genome, gene copies with or without proximity, and certain regulatory elements. They can cause mutations at targeted sites in the genome and specific genetic combinations. This review shows that the potential of NGTs and their special features can be explained by the mode of action of the REMs and the enzymatic processes, e.g., by interfering with repair processes in the cells, thus ultimately leading to targeted mutations. These findings can explain why NGTs can make the genome available to a much greater extent than is the case with conventional breeding (see also [68]).

Another crucial aspect is recently developed REMs that expand the capabilities of genome editing [155]. Their specific technical potential, in combination with AI, was also shown to go beyond what is known from conventional breeding [161]. AI allows for searching large databases to identify and generate specific genetic changes, especially in regulatory units [219]. Especially in combination with the new types of REMs, AI opens up a larger design room for previously unknown gene variations. Therefore, ‘fine tuning’ in NGT plants can be expected to increase in the future.

Regarding the potential of conventional breeding and non-targeted mutagenesis, statements such as “the entire genome can be considered amenable to mutations, providing enough effort and time to achieve and select the desirable mutations” [215] can be found in the literature. Indeed, non-targeted mutagenesis with, e.g., EMS is very effective in mutating plant genetic material [220,221,222], and techniques like TILLING can be used for subsequent targeted selection.

However, constraints in breeding are not generally overcome in this way. As shown above, mutations are unevenly distributed in the plant genome, regardless of whether they occur naturally or are caused by non-targeted mutagenesis [6,7,63,74,84,85]. Established EMS libraries often contain multiple mutated alleles for one gene, while other genes may remain unchanged [84,223]. Although every gene at least in *Arabidopsis* can be mutated at least once, 27% of the genes still lack mutations that could impair protein function [222].

In accordance, researchers also report that they did not find particular mutations, such as null alleles [224], or mutations in specific regions of a gene, e.g., autoinhibitory domains [152], even if they screened large EMS populations. The above-described biological mechanisms, factors, and constraints in conventional breeding raise the question of whether it is merely a statistical problem that certain mutations are not found. In any case, the potential to generate novel genotypes (causing specific phenotypes) in any DNA sequence of interest, including specific combinations of genetic changes, cannot be extrapolated from existing EMS library data. What has been demonstrated, however, is that using NGTs, it is possible to obtain desired genotypes that could not be achieved using non-targeted mutagenesis [152,225].

To put it bluntly, genetic engineering can make almost everything happen simultaneously in all species, whereas conventional breeding and evolutionary processes only allow the development of a certain selection of characteristics over time. In order to equate EMS with NGTs, it would therefore have to be demonstrated that both methods can achieve certain gene variations and combinations with similar likelihood. Since this can not be demonstrated and, in light of the current knowledge, it seems to be unlikely, there is a regulatory need to address the differences. In order to come to meaningful results, the regulatory framework for NGT plants should not only address the actual plants derived from NGT interventions but also the biological mechanisms, factors, and constraints in breeding as described above. Therefore, it is argued that new genotypes derived from NGTs, regardless of the number of mutations, should undergo molecular characterization (as a first step in risk assessment) to assess the causes and effects of their specific differences in comparison to the known gene pool of conventionally bred plants.

So far, it is a matter of fact that the products resulting from the application of NGTs evidence the differences also for complex genetic changes, e.g., in wheat with reduced production of α-gliadines [189], rice with less glutelins [191], sugarcane with less lignin [192], larger inversions [226], changed frequency in crossover events [119], or a combination of several genetic changes in regulatory units [154,155]. As Mundorf et al. summarize, there are high hurdles and an extremely low statistical likelihood of conventional breeding, including non-targeted mutagenesis, ever being able to achieve such complex genotypes [11].

In several cases, also involving minor genetic changes not occurring in conventionally bred genotypes [152,153], further research will be needed to fully understand the many factors at molecular and cellular levels that constrain conventional breeding. At the same time, it is necessary to further scan the horizon for new applications and developments of NGTs to find out whether their application can overcome breeding constraints to ultimately identify causes and effects that result in novel genotypes and phenotypes.

As a result, the existing and known genotypes obtained from conventional plant breeding seem to be the appropriate and practical level of comparison for risk assessment and regulation, rather than the assumed, long-term potential of evolutionary processes. This kind of comparison can help identify new genotypes or traits that are new to the environment and ecosystems at the time of their release, which therefore requires a certain degree of risk assessment. It also places the burden of proof on the applicant requesting approval.

However, in the light of current knowledge, there is no doubt that the outcomes of NGT applications in plants can deviate substantially from those of conventional breeding and non-targeted mutagenesis. Many of the resulting NGT plants may be considered new to the environment, similarly to transgenic plants, even if no additional genes were inserted.

### 4.2. Regulatory Implications

The technical potential to create genotypes and phenotypes, which are unknown from previous breeding processes, has regulatory implications. As mentioned above, Quiroz et al. [6] consider the mechanisms in plant cells as constraints in plant breeding, and as the outcome of evolutionary processes that serve genomic stability and adaptability (see also [1]). Therefore, NGT plants that are new to the environment may negatively impact not only plant health, but also ecosystems and biodiversity. In addition, if these NGT plants are introduced into the environment without sufficient risk assessment, and thus into the breeders’ gene pools, further crossings may reveal unintended and adverse effects not present in the original events [162].

In short, genetically engineered plants with (intended or unintended) characteristics that are new to the environment will need adequate risk assessment before approval, especially if they may be released at a large scale [162]. On the other hand, some NGT plants may show genotypes and phenotypes similar to those obtained from conventional breeding [212]. Therefore, the actual risks of NGT plants may differ case by case. Consequently, there is a requirement for adequate regulation that is not an undue burden on the applicant but nevertheless foresees in-depth risk assessment of NGT plants with novel genotypes and phenotypes. It also has to be acknowledged that different outcomes between NGTs and conventional breeding are not always evident at first sight, nor is their relevance in regard to risk assessment [59]. The differences described here highlight the necessity for a process-oriented assessment. This is in line with the French authority ANSES, which proposes a detailed molecular characterization to identify the need for case-by-case risk assessment [9]. Therefore, the question arises of how adequate regulation can be established to identify the relevant differences in order to protect human health, the environment, plant health, and the breeders’ gene pool, but without generally impeding the potential benefits of NGTs from becoming realized.

### 4.3. Regulatory Concepts

Currently, the European Union is developing a new regulatory framework for NGT plants. In 2023, the EU Commission made a proposal [227,228] that, in late 2025, is still under discussion. As stated in Recital 14 of the Commission proposal [227], NGT plants that could also occur naturally or be achieved with conventional breeding should be treated as such, as they are evidently equivalent and have comparable risks.

Annex I defines five criteria to determine when an NGT plant is considered equivalent to conventional plants (‘Category 1 plants’). These criteria mostly relate to the molecular type (substitution, insertion, deletion, and inversion) and extent of DNA sequence changes.

In its current form, Annex I cannot fulfill its intended purpose for various reasons: Firstly, a threshold value of 20 genetic changes is set, below which modifications are not considered to pose a risk. However, there is no scientific justification for 20 mutations, as, for example, demonstrated by Mundorf et al. [11]. Secondly, genetic modifications are not just characterized by their type and extent, but at least equally by their genomic context, i.e., their location in the genome. Thirdly, limiting the comparison of NGTs and conventional breeding to the type and extent of DNA sequences excludes considering potentials and constraints of both to obtain certain genotypes and decades of practical experience in plant breeding. All these aspects are important to assess the impacts of genetic engineering.

As a result, these criteria mostly relate to the molecular type and extent of DNA sequence changes. As Mundorf et al. state, “Thereby, the criteria follow a paradigm in which genetic changes are isolated from their genomic context and functionality. Accordingly, the proposal neither considers intended and unintended effects nor their phenotypic or risk related outcomes” [11].

It can be concluded that such criteria are not a valid scientific basis for the proposal. They do not fulfill the requirements of Recital 14 to ascertain if an NGT plant is equivalent to naturally occurring or conventionally bred plants by establishing objective criteria based on science. Neither is it in line with proposals for case-by-case decision-making in regard to similarity or dissimilarity, as proposed by EFSA [213], the French agency ANSES [9], or as proposed by the Swiss (https://www.news.admin.ch/de/nsb?id=104720 (accessed on 22 November 2025)) or Norwegian (https://www.stortinget.no/no/Saker-og-publikasjoner/Saker/Sak/?p=102771 (accessed on 22 November 2025)) legislators.

Current EU GMO regulation (Directive 2001/18) requires the examination of all intended and unintended genetic changes with regard to risks to health and the environment. These requirements could be specified in the case of NGT plants. Previous genetic engineering methods typically resulted in the generation of transgenic plants. However, now, in the case of NGTs, plants may be generated that share similarities when compared to conventionally bred plants.

Therefore, based on our above findings, the future regulation of NGT plants should be process-oriented, starting with molecular characterization as a first step in risk assessment to verify differences and similarities of NGT plants in comparison to already known genotypes of conventionally bred plants. For this purpose, methods for genetic analysis and ways to compare the genotype of an NGT plant with its conventional comparators should be defined.

If the result of the molecular characterization does not provide evidence of a new genotype (in regard to intended or unintended genetic changes), then decisions could be taken to reduce the overall amount of data needed for the market authorization of the respective NGT plants.

However, if the NGT plants are found to have a new genotype, further steps must be taken, and more data will be needed to assess the potential impacts on health and the environment. For this purpose, the regulator should define a step-by-step and case-by-case risk assessment that enables robust conclusions on the safety of the NGT plants.

## 5. Conclusions

As shown, the outcomes of the application of NGTs can largely differ from those of previous breeding methods, including physicochemical mutagens. The specific mode of action of the used recombinant genetic enzymes (REMs) is one of the main causes for the differences: While physico-chemical mutagens such as radiation or chemicals merely cause a break in DNA, REMs such as CRISPR/Cas additionally interfere with the cellular repair mechanisms. In addition, more recently developed REMs even expand the capabilities of NGTs to introduce new genetic variations within the target sequences.

Due to these technical characteristics of NGTs, the sites of the induced mutations and their genomic context can differ greatly from the known genotypes and phenotypes obtained from conventional breeding methods, even if no additional genes are inserted.

The differences are crucial not only in terms of the innovations to be expected in plant breeding but also for the risk assessment of the plants in question. The resulting, previously unknown genotypes and phenotypes may negatively impact plant health, ecosystems, biodiversity, and the future of plant breeding. However, some NGT plants may also show characteristics that are similar or even equivalent to plants obtained from conventional breeding.

Therefore, the question arises of how adequate regulation can be established to identify the relevant differences in order to protect health and the environment without placing an undue burden on the applicants. Here, it is suggested to perform case-by-case and step-by-step risk assessment, which should be process-oriented. Similarly to the practice under current EU regulation, the starting point should be a molecular risk assessment, taking into account the intended and unintended effects caused by the respective NGT application. The molecular characterization should take into account not only the type and number of mutations but also their genomic context and the resulting genetic combinations and phenotypes.

Concerning the following steps in the risk assessment and approval process, some flexibility could be established in regard to additional data. This flexibility could be organized on a case-by-case basis according to the degree of similarity or dissimilarity of the NGT plants compared to plant varieties obtained from conventional breeding processes with a history of safe use.

## Data Availability

No new data were created or analyzed in this study. Data sharing is not applicable to this article.
